# Radial Optic Neurotomy: A New Surgical Approach for Glaucoma Treatment?

**Published:** 2012

**Authors:** Ruth E. Rosenstein, Nicolás Belforte

**Affiliations:** 1 Laboratory of Retinal Neurochemistry and Experimental Ophthalmology, Department of Human Biochemistry, School of Medicine, University of Buenos Aires/CEFyBO, CONICET, Buenos Aires, Argentina

**Keywords:** Glaucoma, Optic nerve head, Radial optic neurotomy

## Abstract

Glaucoma is a leading cause of blindness worldwide, characterised by specific visual field defects due to the degeneration of retinal ganglion cells and damage to the optic nerve head (ONH). Elevated intraocular pressure (IOP) is the most important risk factor for glaucoma development. One of the clinical hallmarks of glaucomatous optic neuropathy is the excavation of the ONH, which consists of a progressive posterior displacement of the ONH surface and excavation of the pre-laminar tissues beneath the anterior-most aspect of the scleral canal, known as the anterior scleral ring. Radial optic neurotomy (RON) is a surgical technique that has been proposed for treating central retinal vein occlusion. While the original rationale of RON was the relief of increased tissue pressure within the optic nerve that results from occlusion of the central retinal vein, recent results are discussed here which suggest that by relaxing of the scleral ring of the prelaminar and laminar regions of the ONH, RON may alleviate the IOP-related connective tissue stress, and in turn, prevent the onset and reduce the progression of glaucomatous neuropathy.

## INTRODUCTION

Glaucoma is a leading cause of blindness worldwide, characterised by specific visual field defects due to the degeneration of retinal ganglion cells (RGCs) and damage to the optic nerve head (ONH). Elevated intraocular pressure (IOP) is the most important risk factor for the development of glaucoma. However, the underlying mechanisms that link elevated IOP to RGC death are still not fully understood. The ONH is of particular interest from a biomechanical perspective because it is a weak spot within an otherwise strong corneo-scleral envelope. 

Several lines of evidence suggest that the lamina cribrosa is the principal site of RGC axonal insult in glaucoma [1]. The lamina cribrosa provides structural and functional support to the RGC axons as they pass from the relatively high-pressure environment in the eye to a low-pressure region in the retrobulbar cerebrospinal space [1]. One of the clinical hallmarks of glaucomatous optic neuropathy is the excavation of the ONH, which consists of a progressive posterior displacement of the ONH surface and excavation of the prelaminar tissues beneath the anterior-most aspect of the scleral canal, known as the anterior scleral ring [2, 3]. A considerable body of literature characterised the classic posterior bowing and compression of the lamina cribrosa and excavation of the scleral canal wall beneath the opening in Bruch’s membrane in moderately and severely damaged glaucomatous eyes [4, 5]. Both plastic (permanent) and hypercompliant deformations of the lamina cribrosa and anterior scleral canal wall were described in monkey eyes with early experimental glaucoma [6], which have been confirmed by three-dimensional reconstructions of serially sectioned ONH and peripapillary sclera [7]. It has been postulated that damage to the ONH, the lamina cribrosa, and anterior scleral canal wall connective tissue plays a key role in glaucomatous neuropathy [8, 9]. 

Radial optic neurotomy (RON) is a surgical technique that has been proposed for treating central retinal vein occlusion (CRVO). In 2001, Opremcak et al. [10] hypothesised that CRVO is a compartment-like syndrome which results from increased pressure on the central retinal vein (CRV) within the confined space of the scleral ring. To alleviate this neurovascular compression, the surgical dissection of the lamina cribrosa transvitreally via a radial incision on the nasal side of the optic nerve (ON) (i.e. radial optic neurotomy (RON)) was proposed as a surgical treatment to improve venous outflow. This group reported that 8 out of 11 patients showed an average improvement in visual acuity of five lines after a mean follow-up of 9 months, whereas only two patients worsened [10]. Later on, the same group reported 117 cases treated with RON and 63 cases treated with RON plus an intravitreal triamcinolone injection, in which a gain of two or more lines was shown for 78% and 64% of patients, respectively [11, 12]. Improvements in visual acuity by RON have been confirmed by other investigators [13-17]. However, soon after the proposal of RON as a potential treatment for CRVO, a debate arose regarding whether the incision of the scleral outlet is a reasonable or dangerous procedure [18-21]. The potential risk of nerve fibre defects resulting in visual field loss has been particularly addressed by many authors [18, 19, 22]. Although the radial incision mode and site, as well as the use of microvitreoretinal blades specially designed for RON should minimise vessel and nerve fibre injury, surgical complications cannot be completely ruled out. 

## HYPOTHESIS 

Since the connective tissues of the anterior scleral canal wall are permanently deformed at early stages of glaucoma [6], and considering that the ONH connective tissues are exposed to substantial levels of IOP-related stress/strain [3, 23, 24], it is postulated that by alleviating the constriction of the scleral outlet induced by ocular hypertension, RON could prevent and/or reduce glaucomatous damage. 

## DISCUSSION

Unravelling which are the most critical mechanisms involved in glaucoma is unlikely to be achieved by studies which are limited to the clinically observable changes to the retina and ONH that are seen in human glaucoma. Far more detailed and invasive studies are required, preferably in a readily available animal model. Recently, a model of glaucoma in rats has been developed through weekly injections of chondrotin sulphate (CS) in the eye anterior chamber, which mimics central features of human glaucoma [25]. Thus, this model could be a useful tool for understanding the pathogenic mechanisms involved in glaucomatous neuropathy, as well as for the development of new therapeutic strategies. Using this experimental model, it has been recently shown that RON, which shows no effects per se, decreases functional and histological alterations induced by chronic ocular hypertension in the rat eye [25]. Notably, the retinal protection induced by RON was independent of ocular hypertension, as shown by the fact that it did not affect the increase in IOP induced by CS injections. In hypertensive eyes, a significant decrease of retinal (electroretinogram) and visual pathway (visual evoked potentials) function is observed, whereas RON reduces these functional alterations [25]. Moreover, a significant loss of cells in the ganglion cell layer, and a decrease in Thy-1, NeuN and Brn3a (specific markers of RGCs) levels are observed in eyes injected with CS, whereas RON significantly preserves these parameters. Furthermore, RON preserves the ON structure in eyes with chronic ocular hypertension [25]. Therefore, these results indicate that RON reduces functional and histological alterations induced by experimental chronic ocular hypertension. 

As already mentioned, it is still under debate whether RON would be an adequate treatment or a dangerous procedure. Several surgical complications, such as laceration of the central retinal artery (CRA), further reductions in retinal blood flow, peripapillary retinal detachment from the RON site, ON fibre damage, visual field loss, and focal haemorrhagic pigment epithelium detachment, as well as chorioretinal neovascularisation from the RON site, were described [27- 31]. However, it has recently been shown that RON creates a defect in the lamina cribrosa and surrounding scleral ring of the optic nerve in normal rat eyes, without affecting the CRV and CRA, ocular functions, such as visual evoked potentials, and pupil light reflex, whereas a slight and transient decrease in the electroretinogram (ERG) is observed in eyes submitted to RON [32]. Moreover, no significant vitreous haemorrhage or other serious complications are observed in vehicle- or CS-injected eyes submitted to RON, supporting the fact that, at least in rat eyes, RON is a safe procedure. 

The precise mechanisms responsible for the retinal protection against glaucomatous damage induced by RON remain to be established. For the past 30 years, discussion has focused on how RGC axons are damaged within the lamina cribrosa, and controversy has centred on whether IOP (the mechanical hypothesis) or ONH blood supply (the vascular hypothesis) is responsible for ONH axonal damage in this disease. However, consideration of the anatomy of the lamina cribrosa and peripapillary sclera suggests that the classic mechanical and vascular mechanisms of glaucomatous injury are inseparably intertwined [33]. For example, prior to structural damage, purely IOP-related stress could detrimentally affect the blood supply to the laminar segments of the axons through deformation of the capillary containing connective tissue structures. Also, IOP-related remodelling of the extracellular matrix of the laminar beams could limit the diffusion of nutrients to RGC axons in the ONH. Reciprocally, primary insufficiency in the blood supply to the laminar region could induce cell-mediated connective tissue changes that would serve to weaken the laminar beams, making them more prone to failure under previously safe levels of IOP-related mechanical stress [33]. Moreover, local damage at the lamina has been suggested to account for abnormal axonal transport in human and experimental glaucoma [34, 35]. Burgoyne et al. [3] have proposed that the ONH is a biomechanical structure; this paradigm assumes that IOP-related stress (force/cross-sectional area) and strain (local deformation of the tissues) are central determinants for the pathophysiology of the ONH tissues and their blood supply, particularly at high levels of IOP [33]. While the original rationale of RON was the relief of increased tissue pressure within the ON that results from occlusion of the CRV, the results of this study suggest that relaxation of the scleral ring of the prelaminar and laminar regions of the ONH may alleviate the IOP-related connective tissue stress, which, in turn, could prevent the onset and reduce glaucomatous neuropathy, presumably by counteracting ONH connective tissue damage, maintaining nutritional and oxygen supply, and axoplasmic transport at increased levels of IOP. It has been postulated that IOP-related stress and strain induce changes in the tissues that include not only alterations to the load-bearing connective tissues of the lamina cribrosa and the peripapillary sclera, but also to the cellular components of these tissues, including glial cells, endothelial cells, and pericytes, along with their basement membranes and the RGC axons in the ONH [6]. At present, it could not be ascertained whether there is a specific cellular type that is a target for RON, but this issue will be examined in the near future. 

A major weakness of the mentioned results relies on the differences between the rat and human eye. Although some results support the fact that the lamina cribrosa in the rat eye is thin and poorly developed [36], the rat eye was shown to be a good model for ophthalmological studies because its anatomy is similar to that of the human eye. In fact, it was shown that the rat ONH possess an identifiable lamina cribrosa with structural proteins nearly identical to that of the primate [37, 38]. In contrast, the rabbit, chicken, and quail seem to be less adequate models to study the lamina cribrosa, the major problem being the myelinisation of the axons penetrating through the sparsely developed lamina cribrosa into the nerve fibre layer of the retina. This changes profoundly the situation of cell composition and mechanical reactivity in the ONH region. In addition, the retina from these species is avascular, which probably has a major influence on the ONH blood supply as well [39]. Thus, although care must be taken when extrapolating data generated in rodents to humans, the data of the current study provide evidence supporting the beneficial effects of RON on retinal and ON damage induced by chronic ocular hypertension. Due significance should be given to the fact that the ONH, a very delicate and crucial structure of the eye, is dealt with surgically. However, developments in techniques and technology could increase the margin of safety and efficacy of RON in humans, raising the hope that, in the future, benefits of RON against glaucomatous damage could outweigh the risks of this procedure.

## CONCLUSION

Glaucoma, a leading cause of irreversible blindness, is a progressive neuropathy characterised by loss of vision as a result of RGC death and ON axon loss. Increased IOP is considered the major risk factor in glaucoma. Although the current management of glaucoma is mainly directed at the control of IOP, a therapy that prevents the death of RGCs and ON axon loss should be the main goal of treatment. Recent evidences obtained in a reliable experimental model of glaucoma in rats support the fact that RON, which does not affect IOP, not only prevents but also reduces functional and histological alterations provoked by chronic ocular hypertension. Thus, although further studies are required to evaluate the safety and efficacy of RON in humans, it is suggested that RON could be a new surgical tool helping the challenge faced by ophthalmologists treating glaucoma.

## DISCLOSURE

The authors report no conflicts of interest in this work.
